# Supplementary material for: Committee machine that votes for similarity between materials
Source: IUCrJ. 2018 Oct 30;5(Pt 6):830–40. doi: 10.1107/S2052252518013519 (PMC6211525; doi:10.1107/S2052252518013519)
Supplement: Supplementary file 1 [file m-05-00830-sup1.pdf]

IUCrJ

Volume 6 (2019)

Supporting information for article:

Committee machine that votes for similarity between materials

Duong-Nguyen Nguyen, Tien-Lam Pham, Viet-Cuong Nguyen, Tuan-Dung Ho, Truyen Tran, Keisuke Takahashi and Hieu-Chi Dam

V. Supplemental material

The supplemental material shows additional information for the lattice constant prediction experiment (Section 3.2, Experiment 2) of 1541 binary AB body-centered cubic (b.c.c) crystals. The affinity matrix obtained for all materials is shown in Fig.5a, after rearrangement by a hierarchical clustering algorithm [53]. By utilizing this similarity, we could roughly divide all materials in the dataset into three groups: G1, G2, and G3. Due to the large number of materials in the dataset, a principal component analysis (PCA) [1] is performed to investigate the similarity for materials in each group.

Figure 7 revealed that most materials in G1 are constructed from two heavy transition metals e.g Au, Ag, Ru based materials. For a given A element, the L_{const} of the materials remain in the range from 3.0 to 3.5 Angstrom and slightly increase with increasing of the atomic number of the B element. The highest lattice constant values archive with either noble gas-based or alkali metal-based materials. In contrast, the materials in G2 and G3 are constructed from a metal and a non-metal element, e.g. oxide (AlO, NiO), nitride (AlN, NiN), arsenic (AsTa, AsW) etc. Figure 8 shows the L_{const} of the materials in G2 remains constant for the materials sharing the same A element. On the other hand, the L_{const} for the materials in group G3 mainly depends on the electronegativity difference between the constituent elements A and B, see Figure 9. In this group, for a given A element, the L_{const} of materials increases with increasing of the electronegativity difference.

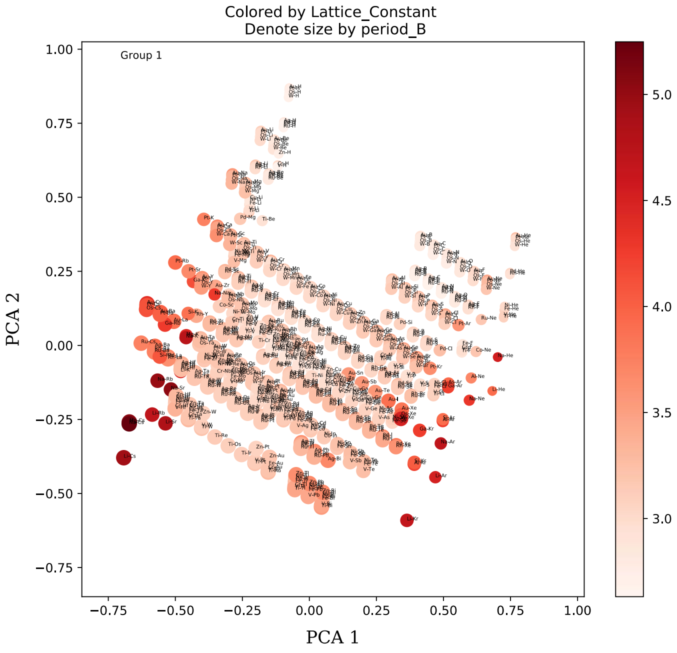

Figure 7: Distribution of materials in G1 for L_{const} prediction problem, shown using two highest variance axes (PCA1 and PCA2) obtained by applying principle component analysis. The colors are assigned based on the L_{const} value, and the material sizes are assigned based on the periods of the B elements.

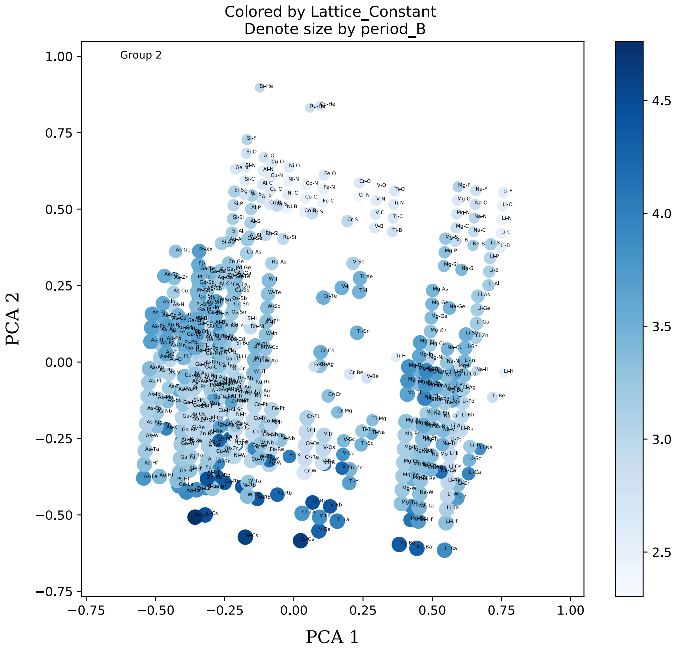

Figure 8: Distribution of materials in G2 for L_{const} prediction problem, shown using two highest variance axes (PCA1 and PCA2) obtained by applying principle component analysis. The colors are assigned based on the L_{const} value, and the material sizes are assigned based on the periods of the B elements.

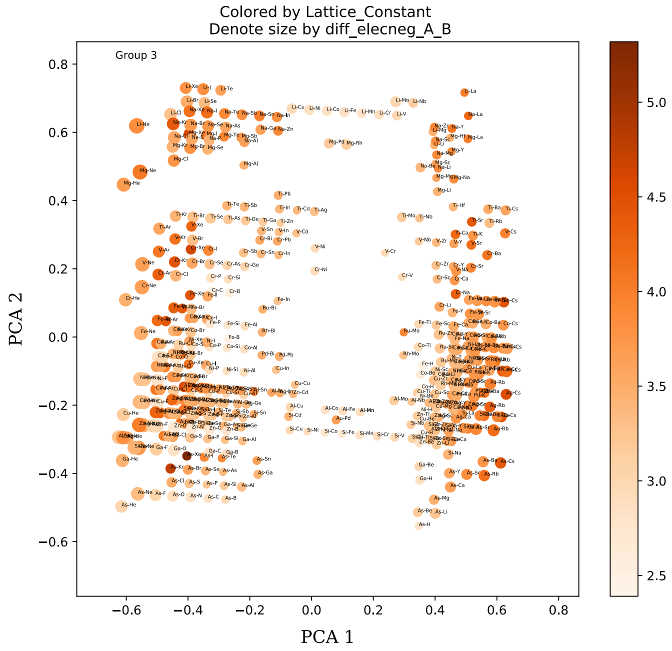

Figure 9: Distribution of materials in G3 for L_{const} prediction problem, shown using two highest variance axes (PCA1 and PCA2) obtained by applying principle component analysis. The colors are assigned based on the L_{const} value, and the material sizes are assigned based on the difference in the electronegativities of the two elements.

[1]: Abdi, H. and Williams, L. J. (2010), Principal component analysis. WIREs Comp Stat, 2: 433-459. doi:10.1002/wics.101
